# The Chieti Affective Action Videos database, a resource for the study of emotions in psychology

**DOI:** 10.1038/s41597-020-0366-1

**Published:** 2020-01-21

**Authors:** Adolfo Di Crosta, Pasquale La Malva, Claudio Manna, Anna Marin, Rocco Palumbo, Maria Cristina Verrocchio, Michela Cortini, Nicola Mammarella, Alberto Di Domenico

**Affiliations:** 1 Department of Neuroscience, Imaging and Clinical Science, University G. d’Annunzio - Via dei Vestini, 31 - 66100 Chieti, Italy; 2 Department of Psychological, Health and Territorial Sciences (DiSPUTer), University G. d’Annunzio - Via dei Vestini, 31 - 66100 Chieti, Italy; 3 Department of Neurology, Boston University, Boston, MA USA

**Keywords:** Human behaviour, Attention, Perception

## Abstract

The Chieti Affective Action Videos (CAAV) is a new database designed for the experimental study of emotions in psychology. The main goal of the CAAV is to provide a wide range of standardized stimuli based on two emotional dimensions: valence and arousal. The CAAV is the first database to present emotional stimuli through videos of actions filmed and developed specifically for experimental research. 444 young adults were recruited to evaluate this database, which consisted of a sub-set of 90 actions filmed in four versions, for a total of 360 videos. The four versions differ based on the gender of the main actor (male or female) and in the perspective in which each action was shot (first-person or third-person). CAAV validation procedure highlighted a distribution of different stimuli based on valence and arousal indexes. The material provided by CAAV can be used in future experimental studies investigating the role of emotions, perception, attention, and memory in addition to the study of differences between gender and perspective taking.

## Abstract (summary)

**Table:** 

Measurement(s)	emotion/affect behavior trait • valence • arousal domain measurement
Technology Type(s)	video
Factor Type(s)	age • sex
Sample Characteristic - Organism	Homo sapiens

Machine-accessible metadata file describing the reported data: 10.6084/m9.figshare.11448795

## Background & Summary

In psychology, a large range of procedures and experimental materials have been used to elicit emotions in a laboratory setting^1–5^. Studies have shown that the use of emotional movie clips could offer additional advantages^6–8^, compared to more simple stimuli. In fact, movie clips present more complex visual stimuli than words and images. Along with being highly capable of involving and capturing the observer’s attention^9^, at the same time, movie clips increase the level of ecological validity, simulating a real-life setting. Furthermore, meta-analyses of emotion induction have shown that movie clips appear to be among the most effective ways to elicit emotions^10^. Despite the advantages of ecological validity and emotional activation, it has been shown that variation in the camera angle and light exposure, within the same movie clip, may lead the observer to perceive some of the real-life situations as unusual or unrealistic^11^. Schaefer *et al*., also has pointed out that most movie clips activate both, the auditory and the visual systems, and that they depict multiple actions using different emotional levels. The multimodal features of most video clips could result in a mixing of different emotional experiences for the observers, confusing the evaluation of the different actions and the recollection of them. Furthermore, the gender of the main actor and the point of view (POV) shot, are not always strictly controlled. The first-person POV allows the observer to take on the perspective of the actor, while in the third-person POV, the observer assumes an external role, watching the action happening. Based on the above rationale, the CAAV (Chieti Affective Action Video) was created with the aim of solving, or at least smoothing-out, some of the issues that affect currently used databases, most of which rely on already existing video clips. Specifically, the CAAV was developed to control for the following critical aspects: the camera angle, the number of stimuli included in the scene, the number of actions presented, the gender of the actors, and the POV with which the actions are carried out.

The most innovative aspect of the CAAV consists in being the first database to present emotional stimuli through videos of actions filmed and developed specifically for experimental research. In relation to the emotional aspect, the development of the CAAV database is founded based on the Dimensional Model of Emotions. This model assumes that it is possible to classify emotions through fundamental dimensions that go beyond the different types of emotional responses^12,13^. This dimensional approach to study emotions is, itself, characterized by the circumplex model^14^, which establishes valence and arousal to be the two dimensions considered when evaluating an emotion. The valence dimension indicates whether the observer likes or dislikes an event, an object, or a situation. The arousal dimension refers to the level of psychological activation induced by an emotional stimulus. According to the circumplex model, from the linear combination of different levels of valence and arousal (e.g. high/low), it is possible to organize different discrete emotions. Specifically to the CAAV database, an example of an action with high level of valence would be “Finding cash”, while one with low level of valence would be “Poisoning a person”. Regarding arousal, an example of an action of the CAAV with high level of arousal would be “Being threatened with a knife”, while one with low level of arousal would be “Sharpening a pencil”. Research, based on this theoretical framework, has led to the development of databases, which has categorized different types of emotional stimuli, such as pictures or words, on the basis of these fundamental dimensions of emotions^1,2^. Therefore, based on this approach, experimental subjects were asked to rate the videos of the CAAV on both, valence and arousal, dimensions. Regarding the CAAV characteristics, controlling for the gender of the actors allows for this database to be used to investigate the role of gender identification. Researchers suggest that gender plays a crucial role in processes related to self-perception, face-recognition, emotion, and memory^15–19^. Furthermore, the perspective dimension (POV) is a critical feature in the present database, since the research participant will either watch the actions being executed by someone else (third-person), or will watch them being performed in first-person as if he/she were performing them. The perspective from which an action is viewed has a fundamental role during the imitation of a behavior, thus influencing the way through which the sensory information is transferred between the person performing an action and the imitator^20^. Several imitation studies have reported that sensory information available from the first-person POV, as if the imitator were observing the model from his/her own perspective, is greater than that viewed from the third-person perspective, where the model is facing the observer^21–23^. The first-person perspective model facilitates more accurate imitative behavior than the third-person perspective model^24,25^ and it induces greater activity in the mirror neuron system (MNS), which is implicated in the processing of visuomotor information^26,27^. Differently, studies investigating the first and third-person through videogames, have highlighted that the playing view influences the presence of emotional responses^28^. The first-person playing view seems to generate a greater emotional response compared to the third-person playing view. Furthermore, participants are more immersed in a game when they look at it through the eyes of the character (first-person condition), regardless of their preferred perspective^29^. Considering all the possible advantages that the use of the first-person POV can have on the observer; our movie clips were filmed using both, the third-person and the first-person point of view. Staging these two different perspectives allows to compare and analyze the different emotional aspects arising from both POVs. Finally, in relation to identification processes and emotional responses, the perspective dimension may further interact with the gender. Therefore, using the CAAV also allows to compare and analyze the mixed emotional aspects arising from the manipulation of these two variables.

## Methods

### Participants

444 healthy participants took part in the CAAV validation procedure voluntarily. Specifically, the sample consisted of 217 young males and 227 young females between the ages of 18 and 30 years (mean = 22.58 years; SD = 3.71), most of which were university students at G. d’Annunzio University of Chieti. They received no compensation and signed an informed consent before starting the experiment. IRB approval was obtained by the G. d’Annunzio University ethical committee.

### CAAV stimuli

**Fig. 1:**
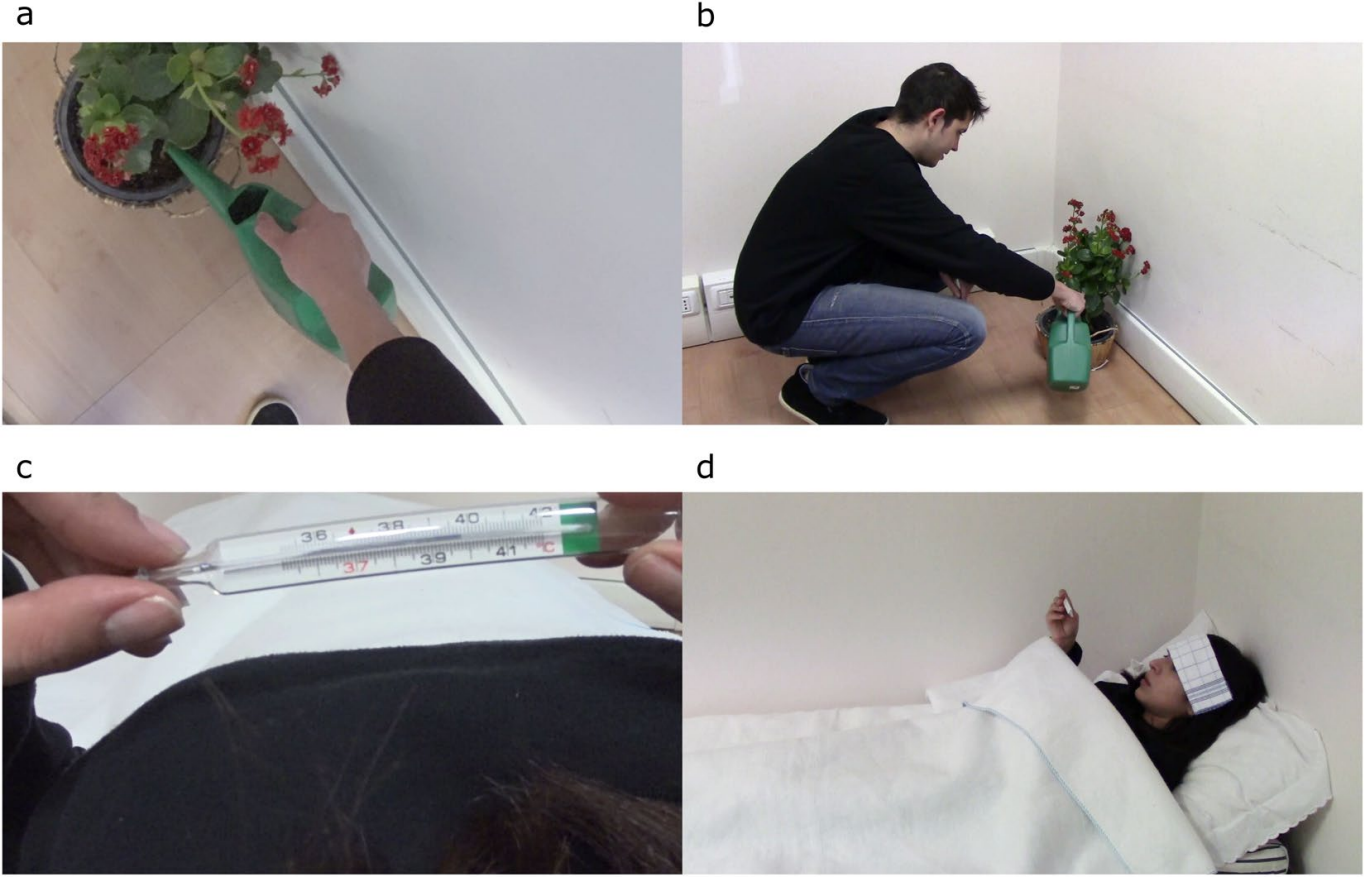
Frames extracted from CAAV videos. (**a**) Action description: “Watering a plant”, first-person POV, male actor. (**b**) “Watering a plant”, third-person POV, male actor. (**c**) Action description: “Measuring one’s fever”, first-person POV, female actor. (**d**) Action description: “Measuring one’s fever”, third-person POV, female actor.

To select the actions to be included in the CAAV we took into account different criteria. Particularly, the actions had to be perceived as realistic as possible. Furthermore, the actions had to be reproducible within a laboratory setting, kept as controlled as possible in order to: (1) avoid presenting too many stimuli simultaneously, (2) avoid variations in camera angle and light exposure, and (3) keep the same setting and background across the videos. Finally, the goal was to select different emotional actions to obtain a subsequent representative distribution of CAAV videos along the valence and arousal dimensions continuum. All CAAV videos were filmed in an empty laboratory room which was kept as controlled as possible. Only if required by the movie clip setting creation, objects were incorporated into the laboratory room, for example the addition of a table, a chair, or a knife. The actions presented in the database were carried out by either female or male characters. Two 24-year-old amateur actors (one male and one female) were involved in the recording, always wearing a black shirt and blue jeans. Videos were recorded with a GoPro Sony HDR-as50. Each video lasted exactly 15 seconds. In order to make all videos the same duration, for actions that were carried out in less than 15 seconds, filler scenes were added (up to a maximum of 5 seconds). In these scenes, the actors were waiting to perform the action. These filler scenes were included to obtain the same standard duration for all stimuli, even though different actions for their own nature were performed by the actors in different time intervals. The CAAV’s emotional stimuli are only depicted through visual presentation. All the auditory stimuli have been muted from the movie clips. In addition, the CAAV presents just one main action in each video, avoiding the succession of many actions that could carry a different emotional valence. Furthermore, our videos have been created to represent a broad sample of contents across the entire affective space and to contain simple and easily understandable actions. The same conditions of brightness were ensured across filming sessions. For videos where differences were detected at a later stage, the brightness was modified using “VideoPad Video Editor”. Finally, the camera angle and the distance of the camera from the scene were controlled across all conditions. To get an overview of what has been described, frames extracted from some videos of the CAAV are shown in Fig. 1.

### Normative rating procedure for CAAV

In summary, our database consists of a sub-set of 90 actions filmed in 4 different conditions for a total of 360 videos: (1) first-person POV with male main actor; (2) first-person POV with female main actor; (3) third-person POV with male main actor and (4) third-person POV with female main actor. The resulting 360 videos were divided into 4 different lists (A, B, C, D). Each list contained all 90 actions but varied based on the gender of the main actor and perspective (first-person/third-person POV). The administration of the 4 different lists was balanced between experimental subjects. This subdivision was needed to reduce the subjects’ fatigue due to the excessive duration of the task^1,2^. To further control for fatigue, each participant was given the option to interrupt the task at any time if s/he was feeling tired. None of the subjects asked to interrupt the task. The presentation order of the videos was randomized within each list and each video was rated for arousal or valence after its presentation. Participants were divided into 2 groups (see Table 1). The first group, composed of 211 participants (101 M/110 F; mean = 22.99 years; SD = 3.81), evaluated the videos based on valence. The second group, composed of 233 participants (116 M/117 F; mean = 22.21 years; SD = 3.60), evaluated the videos based on arousal. The two dimensions of valence and arousal served as dependent measures and were measured through the Self-Assessment Manikin (SAM)^25^.

**Table 1:** Study Characteristics.

Subjects	Experiment	Protocol	Data
211	Rating CAAV videos	Measurement of Valence dimension	Online-only Table 1
233	Rating CAAV videos	Measurement of Arousal dimension	Online-only Table 1

### Tool for rating: self-assessment manikin

According to Russell & Barrett^24^, each emotion arises from the linear combination of valence and arousal. The valence dimension indicates whether the observer likes or dislikes an event, an object, or a situation. Valence ranges along a continuum that goes from negative valence to positive valence. Differently, arousal is defined as the physiological activation level. Arousal compares the states of low physiological activation, which can usually occur in conditions of sadness and relaxation, with those of high physiological activation which can usually be observed in conditions of anger and happiness. In order to measure the emotional dimensions of valence and arousal in relation to our action video clips, we used the Self-Assessment Manikin (SAM) which is a widely used tool in this research field^30^. The SAM is a non-verbal assessment technique which uses figures to measure the valence and arousal associated with a person’s emotional reaction to a variety of stimuli. To study the valence dimension, we used a version of the 9-point SAM scale^30^. In addition, we considered another version of the 9-point SAM scale to analyze the arousal dimension^30^. Using these tools, the subject can select any of the 9 points on the continuum, with 1 corresponding to the lowest possible rating on each dimension (i.e., negative valence/low arousal) and 9 corresponding to the highest possible rating (i.e., positive valence/high arousal).

## Data Records

All data and videos are available on Figshare platform^31^. Specifically, all CAAV videos are reported in the file .zip named “CAAV_database”. Furthermore, eight sample videos of the CAAV are available for download on the Figshare platform as an overview of the CAAV stimuli. The eight sample videos include two different actions, “Losing hair” (low valence/medium-high arousal) and “Blowing candles”(high valence/medium arousal), both presented in four different conditions (in relation to gender and POV variables). All 360 videos are in.mpg format with a 1920 × 1080 resolution. Additionally, the results of the CAAV validation are reported in a separate Excel file. This file is called “CAAV_dataset” and contains a dataset with the average scores, for both valence and arousal dimensions, for each video. This table is accompanied by a legend that provides a detailed description of all the variables. The table reports the code assigned to each video. Each video was renamed with a corresponding code within the depository. Furthermore, this table reports a brief description of the action contained in each video, the type of perspective used (first-person POV vs. third-person POV), and the gender of the actor (male vs female). The average values and the standard deviation for both valence and arousal dimensions are also reported. Furthermore, the mean ratings and standard deviation distinguished by the gender of the experimental subjects (male vs female) are also reported. Finally, in a second Excel file named “CAAV_rawdata” the raw data for all experimental subjects are reported. The table contains: subject ID, age, gender, list of stimuli administered, and the rating for each of the 360 videos.

## Technical Validation

**Fig. 2:**
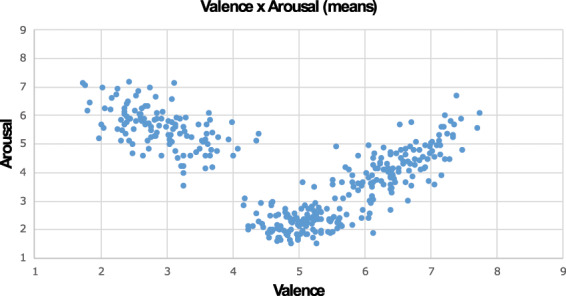
Scatterplot of the interaction between valence and arousal scores of each video. The average valence score is reported on the X axis, while the average arousal score is reported on the Y axis for each video.

In the present study, participants could select any of the 9 points on the SAM rating scale by pressing the corresponding numeric key on a laptop keyboard. Experimental sessions were conducted individually in a laboratory room under similar lighting conditions. Each trial began with a preparatory slide (“Please rate the next video”) that was presented for 3 seconds. Then, the video was presented for its entire duration (15 seconds), and immediately after the video terminated, a new slide with rating instructions was shown. For the first group, instructions stated “Please rate the video based on valence” while for the second group, instructions stated “Please rate the video based on arousal”. After the video disappeared from the screen, subjects made their ratings of valence or arousal using SAM. A standard 5 seconds rating period was used. In addition to the 90 CAAV videos, 3 practice videos (with different randomized perspective and different randomized gender of the actor) were showed prior to the experimental block (“play with a balloon”, “waving a fan” and “punch a wall”). The purpose of these preliminary videos was to train the subjects for subsequent evaluations, informing them of the type of content they would have been exposed to, as well as to familiarize over the use of the rating scales. A laptop computer using E.Prime 2.0 software was used to control the randomization and timing of the stimuli presentation, as well as of the collection of the rating responses. The entire task lasted around 35 minutes. “CAAV_dataset” shows the mean ratings. The interaction between valence and arousal scores of each video is reported in Fig. 2.

## Usage Notes

The present database has the potential to be applied to several fields in psychology. Specifically, since the CAAV stimuli are indexed on both, valence and arousal dimensions, they can be used in experimental studies to investigate the role of emotions. Moreover, the CAAV stimuli can be suitable in cognitive studies to investigate perception, visual attention, and emotional memory. In social psychology, this database and its stimuli can be potentially useful to study morality, responsibility, and empathy. The CAAV can also be used with clinical populations. In this context, for example, video stimuli could be implemented in both emotional and memory training or to investigate reality monitoring and memory distortion. Also, considering further features of the CAAV, it is possible to use this database to investigate the differences related to gender and to perspective taking. Another possibility would be to manipulate these two variables to study the different levels of self-identification and the consequent emotional response. In relation to the limitations and the possible future developments of the CAAV, video stimuli could also be evaluated for other interesting attributes, such as the frequency and typicality of the action in daily life. Finally, since it has been shown that emotional stimuli are processed differently during the life span^32^, a further development could be to involve different age groups in the rating of the CAAV stimuli making the database more appropriate in studies on emotions and memory in aging.
